# Using transjugular intrahepatic portosystemic shunt as the first‐line therapy in secondary prophylaxis of variceal hemorrhage

**DOI:** 10.1111/jgh.14761

**Published:** 2019-07-18

**Authors:** Jiacheng Liu, Qin Shi, Shuping Xiao, Chen Zhou, Binqian Zhou, Feng Yuan, Chuansheng Zheng, Shan Lin, Kun Qian, Gansheng Feng, Bin Xiong

**Affiliations:** ^1^ Department of Radiology, Union Hospital, Tongji Medical College Huazhong University of Science and Technology Wuhan China; ^2^ Department of Ultrasound, Union Hospital, Tongji Medical College Huazhong University of Science and Technology Wuhan China; ^3^ Hubei Province Key Laboratory of Molecular Imaging Wuhan China

**Keywords:** first‐line therapy, portal pressure gradient, secondary prophylaxis, transjugular intrahepatic portosystemic shunt, variceal hemorrhage

## Abstract

**Background and Aim:**

This study aims to evaluate and compare the survival and other portal hypertension‐related complications of patients with portal pressure gradient (PPG) ≥ 25 mmHg using transjugular intrahepatic portosystemic shunt (TIPS) as the first‐line and second‐line therapies in secondary prophylaxis of variceal hemorrhage.

**Methods:**

Fifty patients diagnosed with liver cirrhosis were enrolled in this retrospective study, with 35 of whom received TIPS as the first‐line therapy in secondary prophylaxis of variceal hemorrhage and 15 of whom as second‐line treatment. We observed and analyzed the survival, occurrence of variceal rebleeding and hepatic encephalopathy (HE) of patients in the two groups during the follow up.

**Results:**

The technical success rate was 100%. In a median follow‐up time of 12 (1–37) and 15 (2–27) months, respectively, significant statistical difference was observed between the first‐line group and the second‐line group concerning cumulative survival rate (94.3% *vs* 66.7%, log–rank *P* = 0.01). But that was not the case when it comes to the cumulative rate of variceal rebleeding (8.6% *vs* 26.7%, log–rank *P* = 0.164) and HE (22.9% *vs* 20.0%, log–rank *P* = 0.793). And multivariate analysis indicated that group assignment (hazard ratio = 8.250, 95% confidence interval = 1.383–49.213, *P* = 0.021) was the only predictor of survival. Interestingly, we found that spleen diameter (hazard ratio = 0.578, 95% confidence interval = 0.393–0.849, *P* = 0.005) could be regarded as independent predictor of the occurrence of HE.

**Conclusions:**

For patients with PPG ≥ 25 mmHg who have recovered from an episode of acute esophageal variceal hemorrhage, utilizing TIPS as the first‐line therapy to prevent rebleeding is demonstrated effective in improving the survival and therefore should be recommended to a wider range of clinical practice.

## Introduction

Patients with cirrhosis who have recovered from an episode of acute variceal hemorrhage (VH) are in the state named secondary prophylaxis of VH. If these patients are in high risk of death (combined with VH and other decompensated events), treatments should be concerned with the goal of improving survival rates.^1^ Currently, the first‐line therapy of secondary prophylaxis is mainly composed of nonselective beta blocker (NSBB) + endoscopic variceal ligation (EVL), yet TIPS is only considered as a choice when the first‐line therapy failed to prevent recurrent VH.^2^

As shown by researchers previously, hepatic venous pressure gradient (HVPG) ≥ 20 mmHg (HVPG is generally considered equivalent as portal pressure gradient [PPG])^3^ predicts higher risk of failure, early rehemorrhage, and mortality following endoscopic therapy to control VH.^4^, ^5^, ^6^ However, propranolol could only lower the level of HVPG by 10.1–23.2%, carvedilol by 18.6–27.7%.^7^ (carvedilol is not recommended in the prevention of variceal rebleeding currently).^2^ Therefore, for patients with PPG ≥ 25 mmHg, neither EVL nor NSBB therapy is effective in reducing the mortality of them, even postpone the optimal timing of transjugular intrahepatic portosystemic shunt (TIPS) implantation.^8^, ^9^ TIPS is well known for significantly decreasing the level of PPG,^10^, ^11^ which is only considered as the treatment of choice when the first‐line therapy failed currently. For patients with PPG ≥ 25 mmHg who have high risk of treatment failure and mortality, however, the primary goal should be reducing the level of PPG, which inspires the idea that maybe we should apply TIPS as the first‐line therapy on this kind of patients to prevent variceal rehemorrhage.

Thus, we collected patients with PPG ≥ 25 mmHg in the last few years and analyzed the assumption that using TIPS as the first‐line therapy for them could conspicuously reduce the total mortality.

## Methods

### Patients

Institutional review board approval was obtained for this study. We conducted a retrospective study based on the patients with cirrhosis undergone TIPS from February 2016 to February 2019 at Wuhan Union Hospital.

The inclusion criteria were esophageal VH caused of cirrhosis, pre‐TIPS PPG ≥ 25 mmHg, and no contraindications for TIPS implantation.

Exclusion criteria were the patients without esophageal VH, pre‐TIPS PPG < 25 mmHg, combined with liver tumor, hepatic encephalopathy (HE), or hepatorenal syndrome, which are diagnosed by criteria described before.^12^, ^13^

Fifty patients were enrolled according to the inclusion criteria and exclusion criteria mentioned previously, and they were assigned to two groups. One group of patients were unable or unwilling to be treated with NSBBs or EVL, who received TIPS insertion as soon as possible from day 6 of the index variceal episode.^14^ That is utilizing TIPS as the first‐line therapy in secondary prophylaxis of VH (*n* = 35), all of them reached hemodynamic resuscitation through the treatments of blood volume restitution, vasoactive drugs, balloon tamponade, or antibiotic prophylaxis after acute VH. The other group of patients received TIPS implantation following NSBB + EVL therapy, which failed to prevent rebleeding, that is utilizing TIPS as the second‐line therapy in secondary prophylaxis of VH (*n* = 15). There exists no difference in the baseline characteristics between the two groups of patients (Table 1).

**Table 1:** Baseline characteristics of patients included in the study

Variables	First‐line (*n* = 35)	Second‐line (*n* = 15)	*P* values
Age (years)	51.8 ± 11.2	48.1 ± 9.1	0.262
Sex (male)	20 (57.1%)	10 (66.7%)	0.481
Etiology			0.539
Hepatitis B virus	20 (57.1%)	11 (73.3%)	
Hepatitis C virus	4 (11.4%)	1 (6.7%)	
Autoimmune liver disease	2 (5.7%)	2 (13.3%)	
Alcohol misuse	4 (11.4%)	0	
Unknown	5 (14.3%)	1 (6.7%)	
Laboratory parameters			
Total bilirubin (μmol/L)	22.2 ± 9.9	19.8 ± 7.1	0.390
Albumin (g/L)	31.7 ± 5.9	33.8 ± 7.1	0.268
Alanine aminotransferase (U/L)	26.2 ± 15.0	22.2 ± 9.4	0.343
Aspartate aminotransferase (U/L)	29.4 ± 10.7	31.8 ± 8.9	0.449
Creatinine (μmol/L)	67.5 ± 21.3	66.7 ± 21.9	0.897
Blood urea nitrogen (mmol/L)	5.27 ± 1.94	5.30 ± 2.54	0.967
Prothrombin time (s)	16.6 ± 1.9	16.6 ± 3.6	0.951
International normalized ratio	1.36 ± 0.19	1.37 ± 0.38	0.932
Hemoglobin (g/L)	79.4 ± 16.5	73.9 ± 15.9	0.283
Platelet count (10^9^/L)	69.3 ± 35.4	60.5 ± 30.7	0.406
Serum Na (mmol/L)	139.0 ± 4.3	139.1 ± 3.3	0.885
Child–Pugh score	7.4 ± 1.3	7.1 ± 1.8	0.474
Child–Pugh class			0.066
A	9 (25.7%)	7 (46.7%)	
B	25 (71.4%)	6 (40.0%)	
C	1 (2.9%)	2 (13.3%)	
MELD score	11.2 ± 2.9	10.5 ± 3.7	0.478
MELD‐Na score	11.9 ± 3.9	11.3 ± 3.9	0.23
Imaging evaluation			
Portal vein diameter (mm)	15.7 ± 2.5	17.3 ± 2.9	0.052
Gastric coronary vein diameter (mm)	6.3 ± 2.5	6.1 ± 2.7	0.704
Splenic vein diameter (mm)	12.0 ± 2.4	13.1 ± 2.7	0.175
Spleen diameter (cm)	17.1 ± 3.2	16.6 ± 2.7	0.572
PVT level†			1.000
Grade 0	27 (77.1%)	13 (86.7%)	
Grade 1	4 (11.4%)	1 (6.7%)	
Grade 2	4 (11.4%)	1 (6.7%)	
Grade 3	0	0	
Grade 4	0	0	
Ascites level			0.722
Non‐ascites	4 (11.4%)	3 (20.0%)	
Slight ascites	16 (45.7%)	8 (53.3%)	
Moderate ascites	5 (14.3%)	1 (6.7%)	
Severe ascites	10 (28.6%)	3 (20.0%)	
Pre‐existing portosystemic shunt	5 (14.3%)	3 (20.0%)	0.451
Pre‐TIPS PP (mmHg)	35.5 ± 3.3	36.8 ± 4.8	0.302
Pre‐TIPS PPG (mmHg)	30.0 ± 3.0	30.5 ± 4.5	0.650
Duration of follow up (months)	11.7 ± 4.4	13.1 ± 6.9	0.396

† PVT, portal vein thrombosis, according Yerdel's grade^32^: Grade 1 (< 50% of the PV with or without minimal extension into the SMV), Grade 2 (> 50% occlusion of the PV, including total occlusions, with or without minimal extension into the SMV), Grade 3 (complete thrombosis of both PV and proximal SMV but the distal SMV is open), Grade 4 (complete thrombosis of the PV and proximal as well as distal SMV).

MELD, model for end‐stage liver disease^33^; PP, portal pressure; PPG, portal pressure gradient; PVT, portal vein thrombosis; SMV, superior mesenteric vein; TIPS, transjugular intrahepatic portosystemic shunt.

### Transjugular intrahepatic portosystemic shunt procedures with assessment of treatment outcomes and follow up after treatment

Indicated by TIPS practice criteria,^15^, ^16^, ^17^, ^18^ TIPS insertion of all the patients were operated by one experienced interventional therapist. An 8‐mm expandable PTFE‐covered stent (Fluency, Bard Peripheral Vascular, Tempe, Arizona, USA) were inserted. The primary end‐point of our study is survival, and the secondary end‐point is the occurrence of variceal rebleeding, HE, and shunt dysfunction.

Each patient was hospitalized for several days after undergoing TIPS insertion. During this period, all the patients were treated with analgesia, anticoagulation, liver protection, and strategies for prevention of HE. Routine serological tests were performed to detect liver and kidney function, blood coagulation function, and blood ammonia level respectively 1, 3, 6, 12, and 24 months after TIPS insertion. In addition, stent patency, ascites, and portal thrombosis were evaluated by computed tomography or magnetic resonance imaging.

### Statistical analysis

Continuous variables are presented as the means ± standard deviation, and quantitative variables are presented as absolute numbers (percentages). Categorical variables were compared using Fisher's exact test or *χ* ^2^ test, and continuous variables were compared with unpaired Student's *t* test. Kaplan–Meier curves and log–rank test were used to evaluate the incidence of survival, variceal rebleeding, and HE. Independent predictors were identified with Cox regression model. A *P* value of less than 0.05 was considered to indicate statistical significance. Data processing and analyses were performed by using IBM SPSS statistics version 22.0 (IBM, Inc., Chicago, IL, USA).

## Results

### PPG change and control of ascites

TIPS implantation was completed in all patients, and the technical success rate was 100%. The Pre‐TIPS PPG of all is 30.2 ± 3.5 mmHg, and the first‐line group is 30.0 ± 3.0 mmHg, second‐line group is 30.5 ± 4.5 mmHg, *P* = 0.650. After TIPS implantation, post‐TIPS PPG of the first‐line group and second‐line group decreased to 11.6 ± 2.6 mmHg and 10.8 ± 2.1 mmHg, respectively. PPG level decreased by more than 20% in all, and a total of 37 patients (74.0%) even decreased to below 12 mmHg after TIPS implantation, which is respectively 24 (68.6%) and 13 (86.7%) in the two groups, *P* = 0.163.

No lethal TIPS‐related complication was observed in all the patients. There were 31/35 (88.6%) patients combined with ascites in the first‐line group and 12/15 (80.0%) in the second‐line group. The amount of patients combined with ascites reduced to 6/35 (17.1%) and 3/15 (20.0%) after TIPS insertion. That is to say, the control rate of ascites is 80.6% in the first‐line group and 75.0% in the second‐line group, *P* = 0.489. During the follow up, shunt dysfunction occurred in only one patient in both groups, which later restored shunt patency after repeated balloon dilatation. So the rate of reintervention is 2%.

### Survival, variceal rebleeding, and hepatic encephalopathy during the follow up

The median follow‐up times for the first‐line and second‐line groups were 12 (1–37) and 15 (2–27) months. The cumulative rate of survival, variceal rebleeding, and HE of patients in the two groups are presented in Table 2. We found significant difference (*P* = 0.009) between the two groups concerning survival, but no difference was found as to the occurrence of variceal rebleeding and HE.

**Table 2:** Results of treatment and complications, including survival, variceal rebleeding, and hepatic encephalopathy during the follow up

Variables	First‐line(*n* = 35)	Second‐line(*n* = 15)	*P*values
Death			**0.009**
0–3 months	0	2 (13.3%)	
3–6 months	0	0	
6–12 months	0	2 (13.3%)	
12–24 months	2 (5.7%)	1 (6.7%)	
Variceal rebleeding			0.143
0–3 months	2 (5.7%)	1 (6.7%)	
3–6 months	0	1 (6.7%)	
6–12 months	1 (2.9%)	1 (6.7%)	
12–24 months	0	1 (6.7%)	
Hepatic encephalopathy			0.946
0–3 months	4 (11.4%)	2 (13.3%)	
3–6 months	2 (5.7%)	0	
6–12 months	1 (2.9%)	1 (6.7%)	
12–24 months	1 (2.9%)	0	

**Figure 1:**
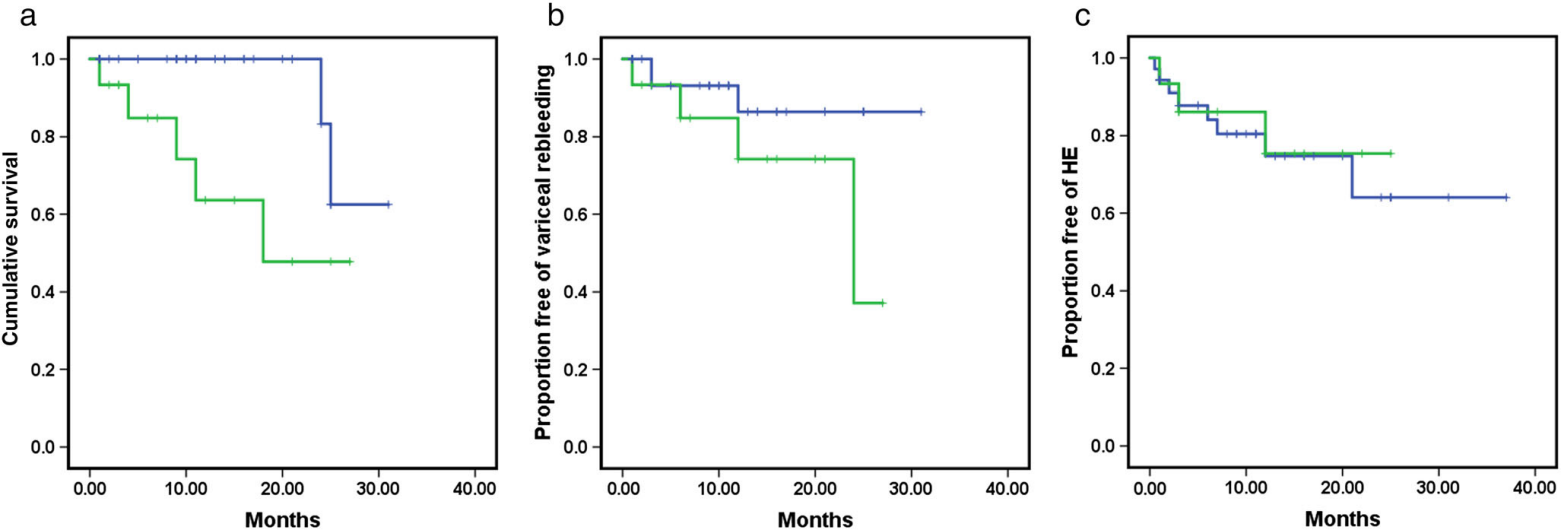
Kaplan–Meier curve of post‐TIPS cumulative survival (a), variceal rebleeding (b), and hepatic encephalopathy (c), the log–rank *P* values of which were respectively 0.01, 0.164, and 0.793. HE, hepatic encephalopathy; TIPS, transjugular intrahepatic portosystemic shunt. (a–c) 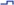, First‐line; 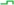, Second‐line; 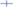, First‐line censored; 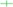, Second‐line censored [Color figure can be viewed at http://wileyonlinelibrary.com]

During the follow up, two deaths were found in the first‐line group: one died of liver failure and the other of upper gastrointestinal rebleeding. In the second‐line group, one patient died from liver failure, three died from upper gastrointestinal rebleeding, and one unknown (Table 2). Demonstrated by Kaplan–Meier analysis (Fig. 1), the cumulative survival rate of the two groups are 94.3% and 66.7%, respectively, log–rank *P* = 0.01. Univariate analysis was conducted according to the survival during the follow up, and we found that patients receiving TIPS as the second‐line therapy and patients with higher pre‐TIPS PP had worse survival. In the multivariate analysis, group assignment (hazard ratio [HR] = 8.250, 95% confidence interval [CI] = 1.383–49.213, *P* = 0.021) was the only predictor of survival (Table 3).

**Table 3:** Univariate and multivariate analysis of factors associated with post‐TIPS outcomes

Outcomes	Univariate analysis			Multivariate analysis		
	Event	Censored	*P* values	HR	95% CI	*P* values
Survival	Dead (*n* = 7)	Alive (*n* = 43)				
Alanine	16.9 ± 5.4	26.3 ± 14.1	0.087	—	—	—
Aminotransferase (U/L)	53.7 ± 6.9	48.4 ± 5.1	0.018	—	—	—
Pre‐TIPS PP (cmH_2_O)	2 (28.6%)	33 (76.7%)	0.020	8.250	1.383‐	0.021
First‐line group					49.213	
Variceal rebleeding	Presence (*n* = 7)	Absence (*n* = 43)				
Prothrombin time (s)	19.1 ± 4.5	16.2 ± 1.9	0.007	—	—	—
International normalized ratio	1.63 ± 0.48	1.33 ± 0.20	0.008	36.357	1.313–1006.488	0.034
First‐line group	3 (42.9%)	32 (74.4%)	0.109	—	—	—
Hepatic encephalopathy	Presence (*n* = 11)	Absence (*n* = 39)				
Albumin (g/L)	29.0 ± 4.6	33.3 ± 6.4	0.045	—	—	—
Prothrombin time (s)	18.4 ± 3.4	16.1 ± 2.0	0.010	1.606	1.062–2.429	0.025
International normalized ratio	1.56 ± 0.36	1.31 ± 0.21	0.010	—	—	—
Spleen diameter (cm)	14.6 ± 3.4	17.6 ± 2.7	0.003	0.578	0.393–0.849	0.005

CI, confidence interval; PP, portal pressure; TIPS, transjugular intrahepatic portosystemic shunt.

After TIPS implantation, variceal rebleeding occurred in a total of seven patients, including three in the first‐line group and four in the second‐line group (8.6% *vs* 26.7%, log–rank *P* = 0.164). Kaplan–Meier curve of rebleeding is presented in Figure 1. In terms of the occurrence of variceal rebleeding during the follow up, the univariate analysis showed that higher prothrombin time (PT) and international normalized ratio (INR) were related. The multivariate analysis showed that only INR (HR = 36.357, 95% CI = 1.313–1006.488, *P* = 0.034) was identified as independent predictors of variceal rebleeding (Table 3).

No patients with HE were found before TIPS implantation in the two groups. However, HE occurred in eight patients and three patients in the first‐line group and the second‐line group following TIPS implantation, respectively (22.9% *vs* 20.0%, log–rank *P* = 0.793). Kaplan–Meier curve of HE is presented in Figure 1. For the time‐to‐event analysis, the following variables including albumin, PT, INR, and spleen diameter were associated closely with HE. According to the Cox proportional hazard model, the independent predictors of HE were PT (HR = 1.606, 95% CI = 1.062–2.429, *P* = 0.025) and spleen diameter (HR = 0.578, 95% CI = 0.393–0.849, *P* = 0.005) (Table 3).

## Discussion

Transjugular intrahepatic portosystemic shunt can significantly reduce the portal pressure in patients with liver cirrhosis and fundamentally alleviate the complications caused by portal hypertension, such as ascites and variceal bleeding. However, on the account that HE and deterioration of liver function are more likely to occur after the implantation of TIPS,^19^ it is only considered as a choice in patients that fail the first‐line therapy (NSBB + EVL) to prevent rebleeding. But this consensus does not seem to be of much reason when it comes to patients with unusual high portal pressure (PPG ≥ 25 mmHg). Some researchers believe that higher reintervention is associated more with TIPS compared with distal splenorenal shunts,^20^ but they used bare stents that are now generally out of use. Instead, expanded polytetrafluoroethylene‐covered stents are recommended by the current criteria of TIPS procedure,^16^ and eloquent evidence has shown that the rate of reintervention can be reduced greatly through regular monitor of expanded polytetrafluoroethylene‐covered stents after insertion.^21^ In our study, shunt dysfunction occurred in only one patient in both groups, which later restored shunt patency after repeated balloon dilatation. The rate of reintervention is 2%, and we believe it is a rather low ratio.

In patients with cirrhosis, the severity of portal pressure correlates to prognosis.^22^, ^23^, ^24^Even treated with nonselective receptor blockers, PPG in patients with variceal bleeding could only be reduced by about 10% and 20% from above 25 mmHg,^7^ which would still be higher than 20 mmHg, and therefore would have a poor chance of survival.^4^ A previous study has proved, in almost half of patients, that NSBBs do not elicit the desired hemodynamic response and do not prevent early rebleeding.^25^And endoscopic therapy as only a means of hemostasis cannot fundamentally reduce the risk of rebleeding.^26^Therefore, we believe that the use of NSBB + EVL as the first‐line treatment in patients with PPG ≥ 25 mmHg not only does not improve the prognosis of patients but also delays the optimal timing of TIPS implantation, only leading to TIPS implantation in the event of uncontrollable rebleeding. For TIPS implantation that can significantly reduce the high PPG of these patients and so can theoretically improve the chance of survival, it is reasonable to be adopted as the first‐line therapy rather than as the second‐line therapy for this kind of patients. We should not choose to overlook TIPS simply for some side effects that actually do not threat the survival of patients.

In this study, PPG decreased by more than 20% in all patients after TIPS implantation, and 74% of whom even fell below 12 mmHg. During the follow up, 2 in 35 patients died in the first‐line group, and 5 in 15 died in the second‐line group; cumulative survival was respectively 94.3% and 66.7%, log–rank *P* = 0.01, suggesting that the survival of the first‐line group was significantly better than the second‐line group. This is due to the earlier application of TIPS, and the great reduction in portal pressure can not only decreases the occurrence of portal hypertension‐related complications but also limits the bacterial translocation and systemic pro‐inflammatory signaling^27^which delays the bad progression and results in better survival for these patients. And as multivariate analysis shows, group assignment was the only predictor of survival, which gives us every reason to believe that TIPS should be considered as a prior strategy for patients with PPG ≥ 25 mmHg, for the risk of death following TIPS as the second‐line therapy was 8.250 times higher than that as the first‐line therapy in secondary prophylaxis of VH.

Because the optimal timing of TIPS implantation has been delayed, mortality was higher in the second‐line group who underwent rescue TIPS after the first‐line treatment failure. The result is consistent with 28, 29In addition, this group of patients has a higher possibility of variceal rebleeding, which may also cause the worse survival. The cumulative variceal rebleeding of the two groups were 8.6% *versus* 26.7% (log–rank *P* = 0.164). Although no significant difference was found in statistics, these data still remind us that TIPS as a second‐line therapy may have higher risk of variceal rebleeding.

In previous researches, the average baseline PPG value in most patients is above 25 mmHg.^31^, ^34^, ^35^So we can speculate that the baseline PPG value of most patients now receiving TIPS as a secondary prophylactic second‐line treatment has already risen above 25 mmHg. That is to say, a lot of them should actually skip the EVL + NSBB treatments and directly undertake TIPS implantation, which may bring them a higher chance of survival and cut down unnecessary medical resources.

Some other interesting outcomes also caught our eye in this research, which illustrated that INR (HR = 36.357, 95% CI = 1.313–1006.488, *P* = 0.034) was identified as independent predictor of variceal rebleeding, and the independent predictors of HE were PT (HR = 1.606, 95% CI = 1.062–2.429, *P* = 0.025) and spleen diameter (HR = 0.578, 95% CI = 0.393–0.849, *P* = 0.005). As to the fact that patients with smaller spleens are more likely to develop HE after TIPS insertion, we reckon this may be due to the more portal vein perfusion in larger spleens, so the extent of portal vein perfusion reduction following TIPS is much lighter than that of smaller spleens under the same circumstance, which as a result lower the incidence of HE. This phenomenon in turn also verifies our proposal that splenic embolization should not be operated at the same time with TIPS implantation. On the other hand, some researchers support the idea that pre‐existing portosystemic shunt (PSS) may be the probable cause for TIPS‐related complications including HE.^30^ And we considered that would also explain our results, as PSS developed smaller spleen, and then involved presence of HE after TIPS insertion. Unfortunately, due to the scarcity of our sample size, we failed to illuminate the relation between PSS and the occurrence of HE.

Finally, we should mention the limitation of our study. First, the lack of a large sample is the major drawback, and further, large‐scale studies are needed. Second, Fluency‐covered rather than Viatorr‐covered stents were used because only the former was available in China. However, it has been reported that the performance of the two stents is similar in preventing variceal rebleeding.^36^Furthermore, what we discussed here was just a retrospective study, and the subjects were not randomly matched. So we are also arranging for a randomized controlled trial that will base on patients with HVPG ≥ 25 mmHg and Child–Pugh score < 10 to confirm the credibility of this study and identify more suitable candidates for TIPS implantation.

In conclusion, for patients with PPG ≥ 25 mmHg who have recovered from an episode of acute esophageal VH, utilizing TIPS as the first‐line therapy to prevent rebleeding is demonstrated effective in improving the survival and therefore should be recommended to a wider range of clinical practice. Furthermore, we found that splenomegaly plays a positive role in preventing HE; hence, it is not recommended to perform TIPS implantation together with partial spleen embolization simultaneously as combined therapy.
